# Forensic medical reporting of non-fatal injuries in criminal cases in the Netherlands: a mixed-methods analysis of regional practices

**DOI:** 10.1007/s00414-025-03678-w

**Published:** 2025-12-18

**Authors:** Maartje L. Goudswaard, Joyce N. Cuijpers, Manon Ceelen, Kim H. de Bruin, Udo J.L. Reijnders, H. Ibrahim Korkmaz, Dionne S. Kringos

**Affiliations:** 1 Department of Forensic Medicine and Medical Advice, Public Health Service Amsterdam, Amsterdam, the Netherlands; 2 Department of Public and Occupational Health, Amsterdam UMC, University of Amsterdam, Amsterdam, the Netherlands; 3 Department of Plastic Reconstructive & Hand Surgery, Amsterdam Movement Sciences (AMS) Institute, Molecular Cell Biology & Immunology, Amsterdam Infection and Immunity (AII) Institute, Amsterdam UMC, Location VUmc, Amsterdam, the Netherlands; 4 Alliance of Dutch Burn Care, Beverwijk, the Netherlands; 5 Burn Center and Department of Plastic and Reconstructive Surgery, Red Cross Hospital, Beverwijk, the Netherlands; 6 Quality of Care, Amsterdam Public Health Research Institute, Amsterdam, the Netherlands

**Keywords:** Injury, Injury examination, Forensic medical examination, Forensic medical injury report, Forensic doctor, Clinical forensic medicine

## Abstract

Non-fatal physical injuries are common in criminal cases, and their accurate documentation and interpretation are crucial for legal proceedings. In the Netherlands, forensic doctors provide independent injury reports that range from basic injury descriptions to translations of medical information into lay terms and comprehensive expert analysis. However, prior research indicates that these reports are often absent in court cases, despite their recognized importance—particularly in serious crimes and domestic violence cases. The reasons for this limited availability remain largely unclear. This study examined the extent and consistency of forensic medical reporting of non-fatal injuries in adults in the Netherlands, identified regional disparities in forensic medical practices, and explored barriers affecting report availability in criminal cases. A mixed-method approach was used, combining a national survey of forensic medical departments with an analysis of injury reports from 2018 to 2022. Findings reveal substantial regional differences in investigation methods, reporting standards, and the number of reports produced. Variations were linked to the lack of requesting protocols, unclear case definitions for forensic doctor involvement, and capacity constraints. The roles of treating physicians, police, and victims in documenting injuries were also not clearly defined. To ensure equitable access to forensic medical expertise within the criminal justice system, this study recommends national standardization, clearer case prioritization for forensic medical involvement, enhanced collaboration, and enhanced forensic training for treating physicians.

## Introduction

Globally, assault and domestic violence are among the most frequently encountered forms of crime. It leads to immense personal, physical and psychological harm. Furthermore, consequences extend far beyond individual suffering, imposing a substantial burden on healthcare systems, legal institutions, and society as a whole [1]. A robust legal response is essential to ensure justice, enable compensation claims, and—particularly in cases of domestic violence—protect victims and prevent further harm. In the Netherlands annually, approximately 40,000 cases of abuse^1^ are registered with the police. Furthermore, Safe Home receives about 24,000 reports of physical (ex-)partner violence yearly [2, 3]. 

Physical injuries frequently play a significant role in criminal cases, as they help determine the cause, severity, and potential fatality risk of offenses. This is particularly relevant in cases of abuse and domestic violence, but also in other offenses such as traffic-related crimes. Objective and independent assessments help prevent misrepresentation or misjudgement in legal proceedings. However, documentation of these injuries for legal purposes varies greatly worldwide, and may involve professionals such as forensic doctors, treating physicians, and forensic pathologists [4]. 

The Dutch inquisitorial system places strong emphasis on written evidence. Legal cases mostly rely on documents such as witness statements, investigation reports, and expert opinions. Oral testimony is limited and less central in trials. Judges actively review the evidence presented in files rather than depending primarily on live courtroom testimony. This approach contrasts with the adversarial systems found in countries like the UK and the US, where oral testimony—especially from expert witnesses—forms a core part of court proceedings [5, 6]. 

In the Netherlands, injury-related documentation to support criminal cases may be provided by several stakeholders. Victims may document their injuries through photographs, while police officers describe, or record them during investigations. Treating physicians can be asked to submit medical files or complete a standardized form describing the injury and the treatment provided. However, because they are not independent and lack forensic training, their role is limited to documenting rather than interpreting injuries [7–9]. 

Forensic doctors perform independent injury examinations—either in person or by assessing case materials such as photographs or medical files. They produce different types of reports. A basic forensic medical injury report typically describes and classifies injuries, whereas an ‘expert report’ provides a detailed interpretation of the findings [10]. Expert reports require formal appointment as an expert. In recent years, forensic medicine has been included in the National Register of Court Experts (NRGD), enabling forensic doctors to register upon meeting the required expertise and training criteria [11]. 

Forensic medical services are organized at both regional and national levels, involving public and private institutions. Regionally, most forensic doctors work within Public Health Services (GGD), except for the private group Forensic Doctors Rotterdam-Rijnmond (FARR). Nationally, the public Netherlands Forensic Institute (NFI) traditionally handles complex cases. Since 2022 a private institution^2^, has additionally been contracted by the Ministry of Justice and Security, due to staff shortages at the NFI. From 2022 it conducts sexual assault examinations in minors, and since 2024, child abuse and adult injury examinations [12–14]. 

The involvement of forensic doctors is generally guided by specific protocols or legal requirements. For non-natural deaths, their role is legally mandated. For sexual assault and child abuse, the established of the Sexual Assault Centers (CSG) in 2012, and the development of the Framework for the Investigation of Child Abuse (FMEK) in 2020, formalised working agreements [15–18]. 

In contrast, formal procedures for documenting non-fatal injuries in adults are less defined. Legal guidelines recognize the importance of forensic examinations. For example, Public Prosecution Service directives on domestic violence stress the importance of injury documentation as evidence, and police instructions require officers to immediately contact a forensic doctor in non-fatal strangulation cases [19, 20]. However, practical implementation is generally unclear.

A challenge in forensic medical practice is a shortage of forensic doctors and limited specialised training. Numerous reports in recent years have highlighted difficulties in service quality and availability [21, 22]. Several initiatives aim to address these issues; through recruitment, enhanced training, and government-funded research [23–25]. This study is part of these nationwide research initiatives to strengthen forensic medicine [26]. 

Previous research from 2008 revealed significant regional variation in forensic medical injury investigations of domestic violence across police regions [27]. Research also highlighted that forensic medical reports are often unavailable in Dutch court cases. An analysis of 24 criminal files from 2016 to 97 published verdicts (2020–2021) from Amsterdam courts found that 10% contained a forensic medical report, additionally 25% included information from a treating physician. In interviews with stakeholders, focus groups with judges, and a survey among public prosecutors, concerns were raised about the absence of forensic medical reports and about the accessibility and quality of information provided by treating physicians. In the absence of forensic medical interpretation, judges often attempt to interpret medical information themselves, despite lacking the necessary expertise. Judges also frequently avoid requesting further information to prevent delays in proceedings [28–30]. 

This study focuses on non-fatal injury cases in adults, excluding minors (e.g., child abuse) and sexual assault, as separate national frameworks exist for these cases. It aims to examine current procedures, practices, barriers, and the number of forensic medical reports across forensic medical departments in the Netherlands.

Specifically, it seeks to answer two research questions: (1) What are the existing working methods and barriers within forensic medical departments regarding non-fatal injury investigation and reporting? (2) What type and how many forensic medical injury reports were compiled by forensic doctors in the Netherlands between 2018 and 2022, and what regional differences exist?

By addressing these questions, the study provides insight into variations among forensic medical departments, and identifies potential areas for improvement. These findings are valuable for advancing forensic medicine and for the potential future application of injury dating methods currently under investigation [26]. 

## Research methods

This study combines a mixed-method approach: a survey targeting forensic medical departments and a comprehensive analysis of injury reports from 2018 to 2022.

### Qualitative survey

#### Study design and data collection

A cross-sectional, regional-level survey was developed in Dutch using LimeSurvey. The survey targeted forensic doctors with expertise in injury reporting, as well as medical and department managers. Recipients were asked to forward the email to relevant colleagues. It was distributed electronically on March 8, 2023, to the managers from all 23 forensic medical departments in the Netherlands. Additionally, an announcement was posted on the website of the Forensic Medical Association (FMG) on March 23, 2023. The survey remained open until the end of April 2023. Clarifications were obtained through follow-up telephone calls with five forensic medical departments.

#### Development of the survey

The survey was collaboratively developed by practicing forensic doctors with experience in injury reporting and researchers providing methodological input. The final version consisted of 15 questions with multiple-choice answers and optional fields for further explanation.

The survey questions covered the following topics:

- Respondent characteristics (region, function).
- Policies within the forensic medical department regarding:
- Request procedures, including agreements with police, judiciary, and guidelines.
- Injury investigation methods (location, timing, method, and duration).
- Role of general practitioners (GPs) in injury reporting and agreements with treating physicians.
- Number of forensic doctors involved in injury reporting and impact of capacity issues on injury reporting.
- Methods for registering injury reports; based on these outcomes a quantitative analysis of the number of reports was conducted.

### Quantitative data

For the period 2018–2022, the number of injury reports produced by forensic doctors was analysed using data from the following sources:

#### The forensic doctor registration system (Formatus)

- Injury description reports: the registration category ‘injury description’ was used across all departments for analysis. Reports involving child abuse were excluded by omitting cases involving minors that were either registered as domestic violence, or requested by child protection organizations.
- Injury translation reports: the registration categories, ‘requesting medical information’ and ‘medical certificate’ were used for analysis, depending on departmental practices. To account for duplicates, multiple entries to the same client within a year were consolidated and counted as a single report.
- Expert reports: the registration categories ‘medical certificate’, ‘miscellaneous’ and ‘injury description’ were used for analysis. However, due to the substantial variability in registration, comparison was not possible.

#### Submitted data

- Forensic Doctors Rotterdam Rijnmond (FARR):

The FARR is the only regional forensic medical department not using Formatus. They provided annual figures for the different report types. Child abuse cases, reported separately, were excluded.

- Netherlands Forensic Institute (NFI):

The NFI only produces expert reports. They provided a database in Excel, including all adult cases from 2018 to 2022 for which a report was requested (total 416 cases). Cases were excluded if they were recorded as rejected with no report produced (63 cases), unrelated to criminal law (25 cases), or not completed within the 2018–2022 period (9 cases). This resulted in a total of 319 completed reports within 2018–2022.

### Data analysis

#### Grouping of data

**Fig. 1:**
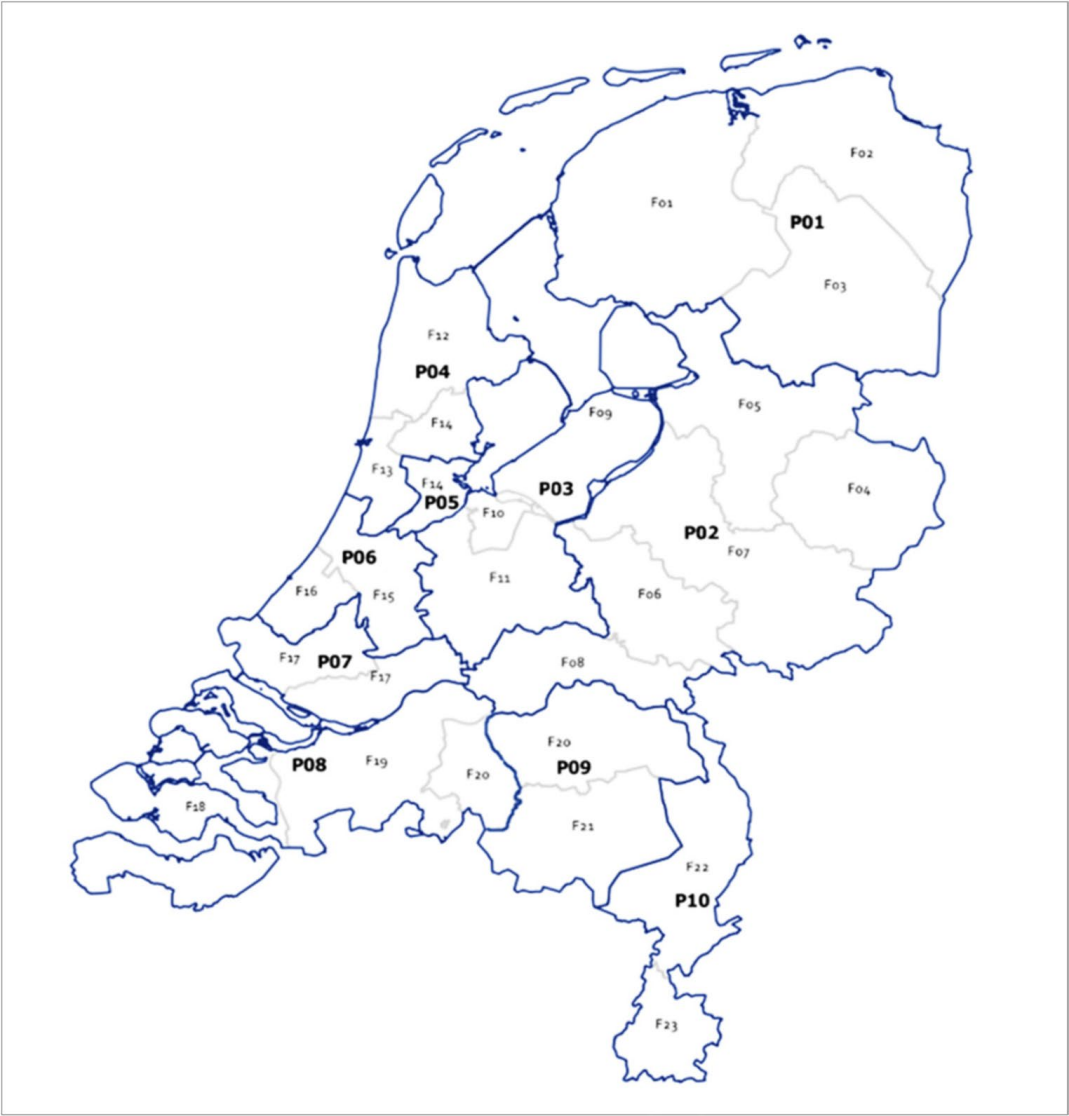
Map of the Netherlands. The map presents the forensic medical departments (F01–F23), which operate within the 25 Public Health Service (GGD) regions (outlined in grey), as well as the police regions (P01–P10) (outlined in blue). The names associated with these identifiers are listed in Figure 2

Data from the regional forensic medical departments (F01–F23) were grouped according to police regions (P01–P10). Figure 1 provides the geographical distribution of these identifiers.

**Fig. 2:**
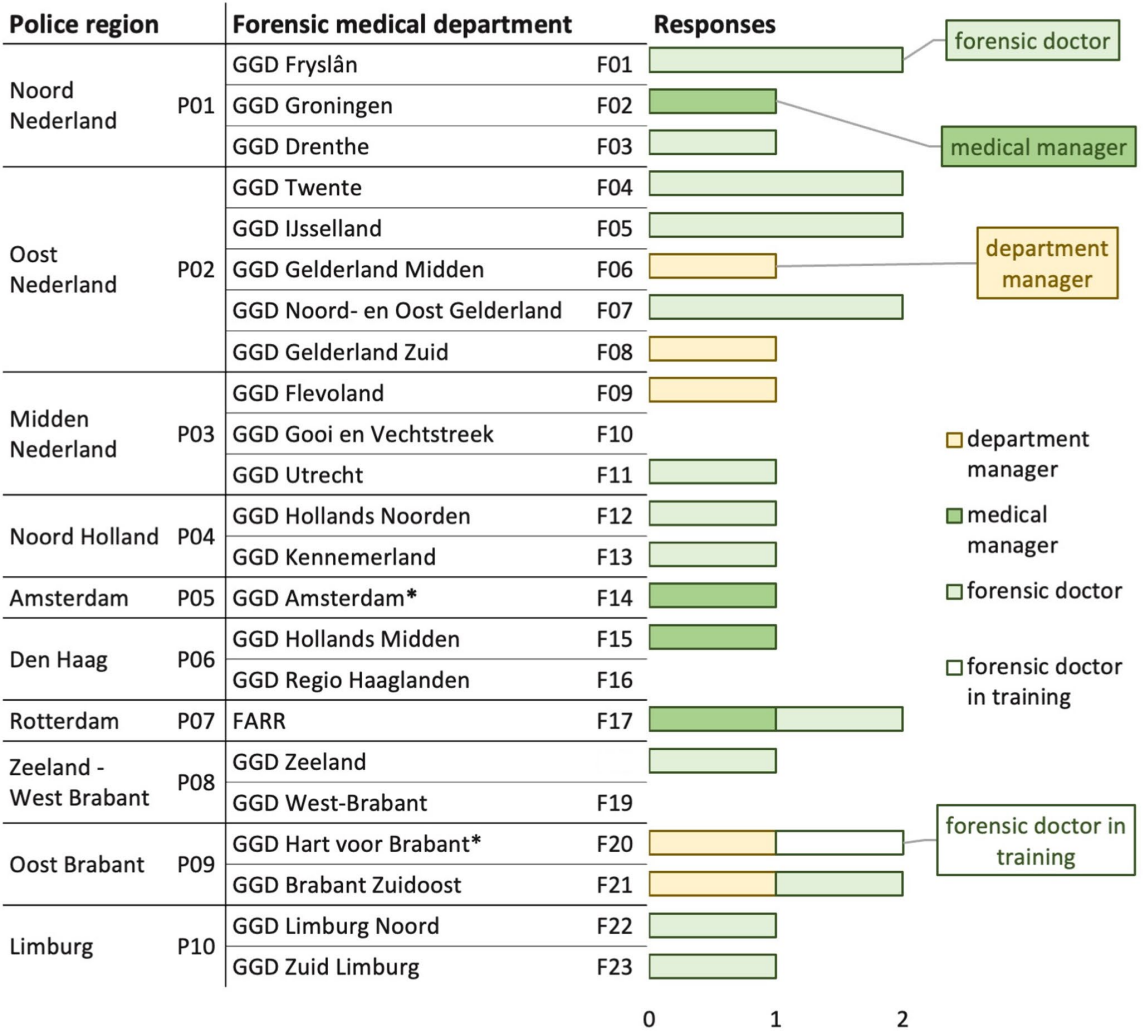
Respondents per forensic medical department and role. Number of responses per forensic medical department (F01–F23) and police region (P01–P10), categorized by respondent role. *F14 and *F20* cover two police regions but are listed under the region with the largest coverage

Data from the NFI were grouped according to the requesting police region (P01–P10).

#### Qualitative survey data

Descriptive statistics were performed on the multiple-choice responses to the survey using Microsoft Excel 2019 MSO and IBM SPSS Statistics 28.0 (2021). A thematic analysis was conducted for the optional explanatory answers, identifying recurring themes and patterns in the open-ended responses.

#### Quantitative data

Descriptive statistics were conducted using Microsoft Excel 2019 MSO and IBM SPSS Statistics 28.0 (2021). The number of reports was related to population size using demographic data from 2018 to 2022 [31].

## Results

### Respondent characteristics (region, function)

The survey was returned by 27 respondents, representing 20 of the 23 forensic medical departments. The respondents included 18 forensic doctors (including one in training), four medical managers, and five department managers. Each department had a response rate of 1–2 submissions. At least one response was received from each police region. Figure 2 presents the number of responses received, categorized by forensic medical department and respondent role.

### Request procedures

Respondents reported considerable variation in request procedures for forensic medical investigations. Requests were initiated by the victim or police, and submitted via email, telephone, direct contact with forensic doctors, or through the secretariat. Examinations were either scheduled by appointment, conducted ad hoc by doctors during shifts, or assigned to dedicated doctors at specialized consultation services.

Only three forensic medical departments reported having formal protocols documenting working arrangements for injury investigations with the police and judiciary; however these were not shared. Thirteen departments indicated an absence of such protocols, with some noting informal agreements or ongoing protocol development. One department stated that forensic doctors are involved only in cases of ‘more serious injuries,’ though this was not explicitly defined.

### Injury investigation methods

**Fig. 3:**
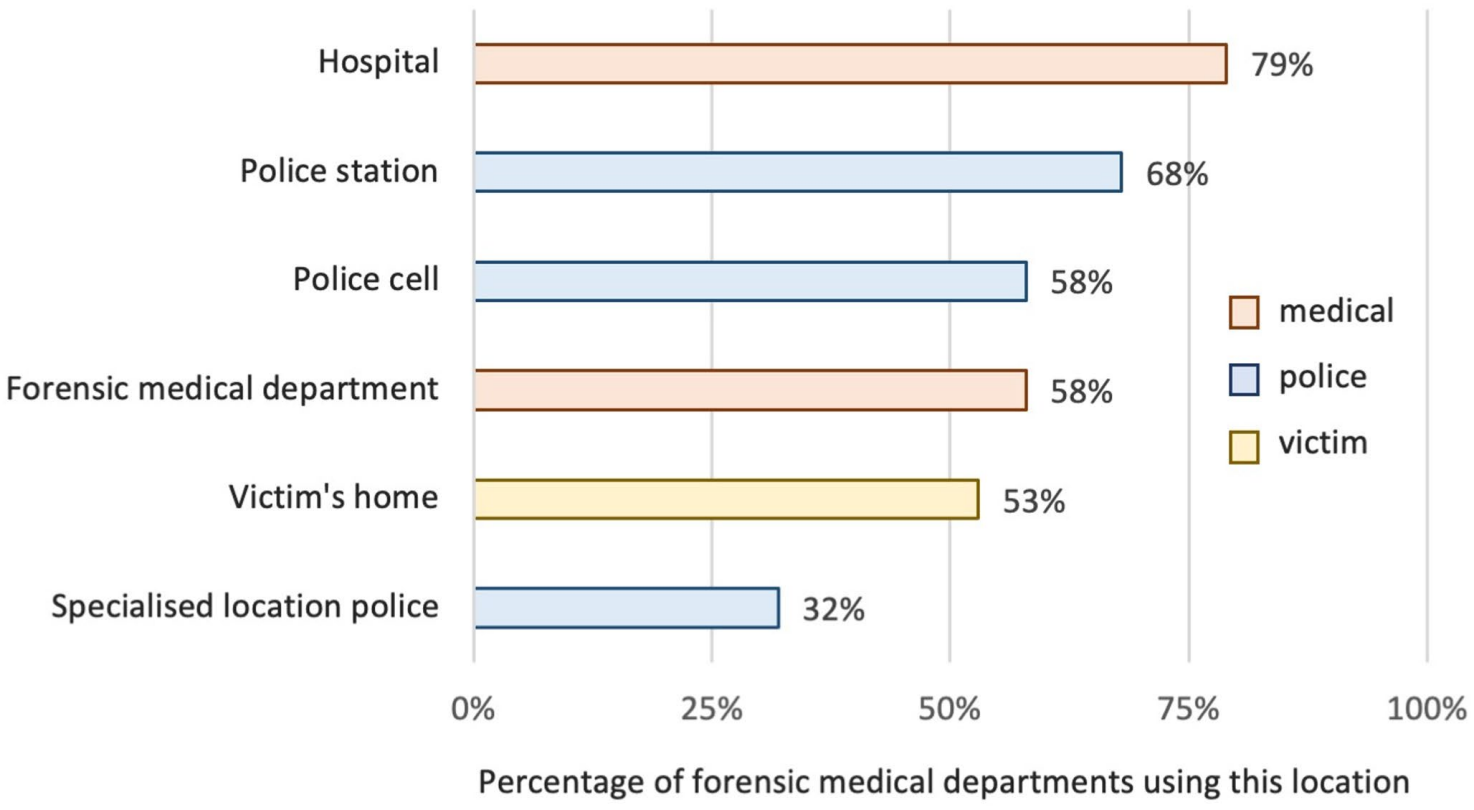
Use of injury examination locations. Percentage of forensic medical departments conducting examinations in each type of location

Forensic doctors conducted injury examinations of victims or suspects at up to six different locations, with travel times ranging from 10 to 90 min. The most frequently reported examination location was the hospital, followed by the police station, the forensic medical department, police cells (for suspects), the victim’s home, and, least frequently, a specially designated location within the police department. Figure 3 shows the percentage of departments using the different locations.

Most departments conducted all four reported types of examinations: injury examinations performed independently or with the police forensic services, photograph-based reviews, and medical record assessments. Four departments reported that forensic doctors never conducted injury examinations independently but always worked alongside the police (forensic services) for photographic evidence. In two other departments, medical records were not reviewed at all.

Explanatory comments indicated that forensic doctors usually performed injury examinations independently in less serious cases. While in more serious cases, collaboration with police forensic services for photographic evidence was common. The distinction between less and more serious cases was not specified. Photo-based examinations were commonly used in minor injury cases, when injuries were no longer visible, when a physical examination was not feasible, or in cases involving abuse against officials (e.g., police officers). The images were typically taken by the police, the victim, or in a hospital prior to medical treatment. Reviews of medical records were frequently used in more serious cases and particularly common in traffic-related offenses. Again, the classification of minor injuries or more serious cases was not defined.

Different formats were used for the injury description reports. Some followed the format generated by the forensic registration system (Formatus) based on input fields. Others used separate documents based the Forensic Medical Association format or on a self-developed template. The format also differed within departments, depending on the doctor’s preference. Report completion times ranged from 1 to 8 h for injury description reports and 1 to 35 h for expert reports.

### Role of general practitioners (GPs)

One forensic medical department indicated that GPs in their region produced injury description reports, while six departments reported that GPs in their regions did not. In other departments, this was unclear. Explanatory comments noted that GPs and other treating physicians typically filled in ‘police notes’. Additionally, five departments reported having agreements with treating physicians regarding injury reports, though none provided these protocols. Twelve departments indicated no such agreements, while others were uncertain.

### Number of forensic doctors and capacity issues

The number of forensic doctors per department varied, with an average of 9.7 and a range of 4 to 21. In most participating forensic medical departments (17 out of 20), all forensic doctors were involved in producing injury description reports. Expert reports were not produced by all departments and were handled by a limited number of forensic doctors, up to five per department. A maximum of two doctors per department were registered in the National Register of Court Experts (NRGD). Some doctors worked across multiple regions.

One respondent indicated that their department had stopped producing expert reports due to shortages, six respondents indicated that their department sometimes or regularly had to decline requests for injury description reports and/or expert reports due to a shortage of forensic doctors. Responses from one department were conflicting. One respondent reported declining requests, while another stated that, to their knowledge, all requests could be fulfilled—although sometimes with longer lead times than the police preferred. Other departments similarly reported increased lead times and longer waiting times due to shortages.

### Methods for registering injury reports and number of reports

#### Regional forensic medical departments

Based on survey responses and data analysis, the regional forensic medical departments produced three types of reports:

1. injury description reports typically include an injury description and classification,
2. injury translation reports consist of a translation of the medical records,
3. expert reports provide a comprehensive interpretation of the injuries.

With the exception of FARR (F17), which uses a different registration system (the GP information system MicroHIS), all regional forensic medical departments use the forensic doctor registration system (Formatus).

In Formatus, different registration methods were used:

4. injury description reports under ‘injury description’,
5. injury translation reports under ‘requesting medical information’ or ‘medical certificate’.
6. expert reports under ‘medical certificate’, ‘miscellaneous’, ‘injury description’ or not registered at all.

The number of reports varied across regional forensic medical departments. The average annual numbers for 2018–2022 are presented by report type:

7. All regional forensic medical departments produced injury description reports, totalling 2,225 annually. This corresponds to an average of 106 reports per department annually (range 4–659; median: 44). On average, 19% of these reports involved domestic violence cases (range 12%-32%).
8. Four departments produced injury translation reports, totalling 2,062 annually. This corresponds to an average of 516.
  reports per department annually (range: 55–1,570; median: 219).
9. Due to inconsistent registration practices, the number of expert reports doctors could not be determined. Estimates from nine departments ranged from 0 to 20 reports per year.

#### NFI

**Fig. 4:**
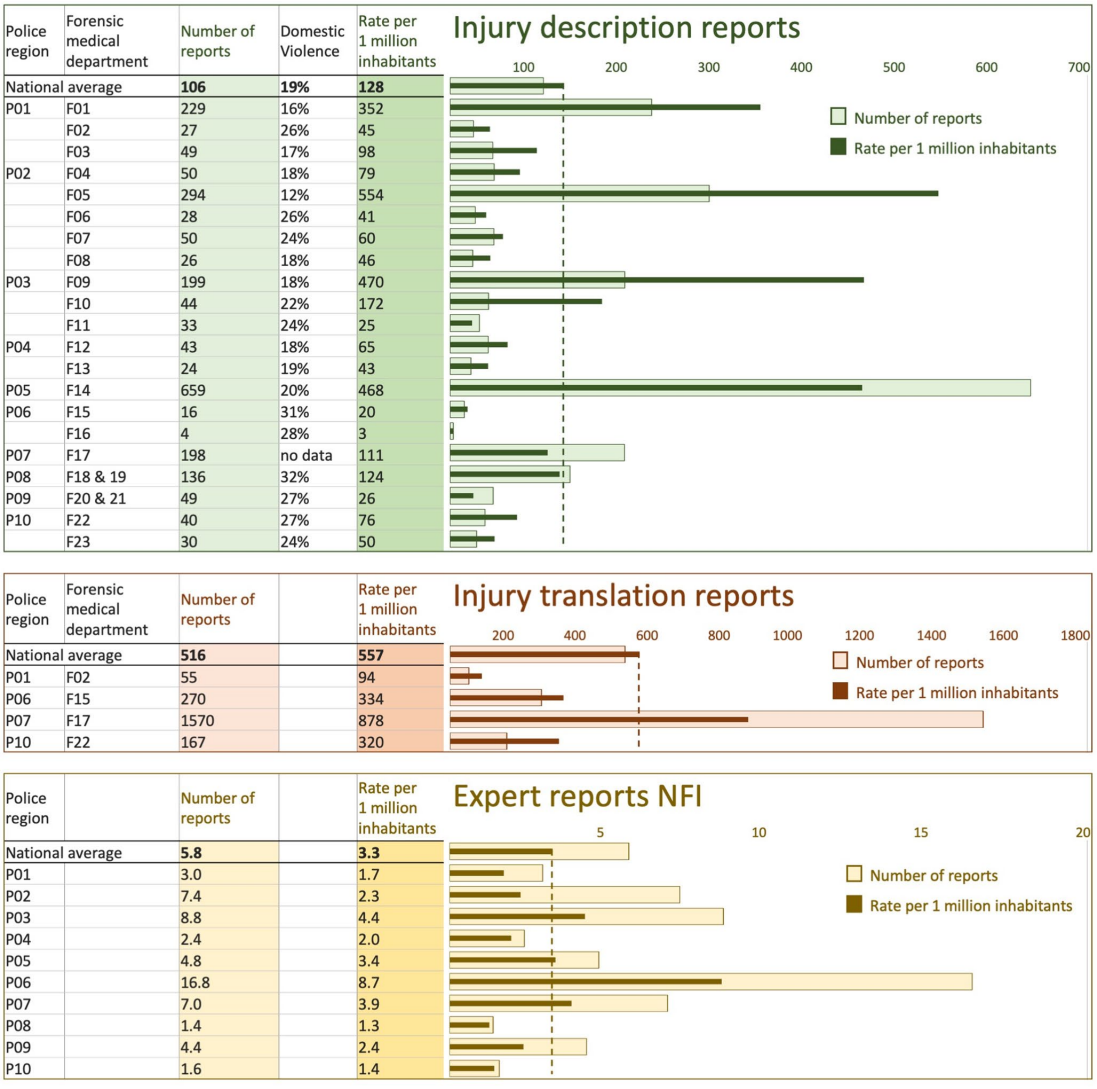
Injury and expert reports. Average annual number of injury description, injury translation, and expert reports (2018–2022) per forensic medical department and police region, shown with population-adjusted rates per 1 million inhabitants. Lighter bars represent annual numbers; darker bars indicate population-adjusted rates. Dotted lines represent national averages

The NFI produced an average of 64 expert reports annually between 2018 and 2022. After excluding 31 of the 319 reports that could not be assigned to a specific police region, this equates to an average of 5.8 reports per requesting police region per year (range: 1.4–16.8; median: 4.6). Figure 4 presents the annual number of reports per forensic medical department and police region, as well as the rate per one million inhabitants to account for population differences.

## Discussion

This study was part of a government-funded initiative to advance forensic medicine in the Netherlands. Specifically, it explored the potential for implementing novel injury dating methods, which require clear procedures and standardized protocols for forensic medical injury investigations [25, 26]. Furthermore, it was warranted by previous research highlighting that forensic medical reports are frequently absent in court cases involving non-fatal injuries in adults, despite their recognized importance for judicial decision-making [28, 29]. It examined current procedures, practices, barriers, and the number of forensic medical reports related to non-fatal injuries in adults across forensic medical departments in the Netherlands.

This study revealed substantial regional differences in working methods and procedures, the number of reports produced, capacity constraints among forensic doctors, and the role of the treating physician. These findings suggest underlying challenges and point to several areas for improvement.

Forensic injury investigation and reporting methods varied considerably across forensic medical departments. Some consistency was observed in specific situations—for example, collaboration with the police forensic services for photographic evidence and review of medical records in ‘more serious cases’. However, the criteria for defining a serious case were not specified. Several differences in practices remained, particularly in how findings were reported.

While guidelines distinguish only two types of reports—a forensic medical injury report and an expert report (see introduction)—this study identified three types in practice: injury description, injury translation, and expert reports (see Sect. 3.6) [10]. This additional categorization highlights a lack of standardization in the use of medical records: some departments incorporated these assessments into injury description reports, others issued separate injury translation reports, and some did not review medical records at all. Additionally, different report formats were used, both within and between departments.

These variations appear to reflect a lack of consensus on national standards. Historically, the development of protocols has depended on volunteer efforts from members of the Forensic Medical Association. However, the growing recognition of the need for increased professionalization in forensic medicine has recently led to funding aimed at developing evidence-based protocols [32]. These efforts are likely to support greater consistency and ultimately help standardize the range of services offered. Ideally, the establishment of a professionally structured institute for forensic medicine guideline development would ensure continuous updates and effective implementation of standards across the field.

In addition to differences in investigation and reporting methods, the number of reports produced also varied considerably between regions. After adjusting for population size, four forensic medical departments—GGD Fryslân (F01), IJsselland (F05), Flevoland (F09), and Amsterdam (F14)—generated significantly more injury description reports, while FARR (F17) produced substantially more injury translation reports. These discrepancies appear to be linked to varying collaboration practices between police and forensic doctors and differing degrees of forensic medical involvement in criminal cases. For example, several departments reported that forensic doctors never conduct injury examinations without police presence for photographing injuries, while in most other regions these examinations were also conducted independently. Additionally, some departments had access to specialized facilities within the police, while the majority did not. Furthermore, in some regions, forensic doctors could be involved in all police-reported injury cases, including minor ones, through specialized consultation services. In other regions, in contrast, their involvement was limited to cases of ‘more serious injury’. However, as with the term ‘serious cases’ mentioned in the previous paragraph, the criteria for defining ‘serious injury’ were not specified. It remains unclear, for instance, whether domestic violence cases are classified as ‘serious’ or given priority. Notably, the proportion of reports concerning domestic violence was relatively low, with considerable variation between departments. While existing directives from the police and the Public Prosecution Service, as outlined in the introduction, formally designate these cases as a priority, the findings suggest that such prioritization is not consistently applied in practice [19, 20]. The survey also indicated that formal request procedures or protocols that would clearly define these priorities were generally lacking.

Regional disparities were also observed in the number of expert reports issued by the NFI, with higher per capita numbers in the urban regions of The Hague (P06), Midden-Nederland (P03), Rotterdam (P07), and Amsterdam (P05) compared to other regions. These differences may partly reflect higher rates of serious crime in urban areas, but other factors are likely to contribute as well. For instance, the number of expert reports in The Hague region—where the NFI is headquartered—was approximately twice as high as in regions with other major cities.

Capacity limitations were found to restrict service delivery, resulting in declined requests and extended waiting times for forensic medical examinations. Although substantial differences in the number of doctors were observed across departments, these variations did not appear to directly explain the decline in requests. However, comparison between departments was challenging due to a lack of information on contract types (full-time versus part-time) and because some doctors were employed across multiple regions. Strengthening collaboration between forensic medical departments could support more consistent service delivery. Variations were observed even among departments within the same police region. This suggests that, despite collaboration efforts initiated in 2019, further measures are needed to harmonize services and enhance cooperation within police regions [22]. The ongoing privatization of forensic medical services, introduced to address workforce shortages at the NFI, may complicate staff retention in the public sector. Continued monitoring is needed to ensure these developments support equitable access to forensic medical expertise and contribute to balanced service provision across all regions.

Given the capacity constraints among forensic doctors, a more targeted approach to forensic medical involvement in non-fatal injury appears necessary. Prioritizing cases involving serious indictments and domestic violence, would align with both legal requirements and ethical imperatives. Previous research supports this prioritization, indicating that judges specifically request forensic medical reports in serious offenses such as grievous bodily harm, attempted manslaughter or murder, and domestic and sexual violence cases [28, 29]. High Court jurisprudence on grievous bodily harm further underscores the importance of detailed medical information—such as the nature of medical interventions and recovery prospects—as critical evidence in court proceedings [33]. In addition, the Netherlands’ obligations under the Istanbul Convention emphasize the need for comprehensive investigations into violence against women, requiring adequate forensic medical expertise in intimate partner and sexual violence cases [34]. 

This study found that the Amsterdam region (F14) produced a relatively high number of injury description reports. However, previous research indicated that forensic medical evidence was included in only about 10% of court verdicts in Amsterdam courts [28, 29]. This comparison raises questions about the effectiveness of the current use of forensic medical capacity.

Injury description reports are typically requested shortly after police reporting. In general, only about 10% of reported criminal cases proceed to trial [2, 35]. As a result, many reports are likely produced for cases that do not reach court. It remains unclear whether these reports are used in police investigations or prosecution decisions, and their value at these stages is uncertain.

At the same time, cases that do proceed to court may lack sufficient forensic medical evidence. Injury description reports may not always provide the level of detail required for judicial proceedings, where expert interpretation of injuries is often needed. Expert reports are more detailed and time-consuming to produce but remain limited in number.

These findings suggest a mismatch between where forensic medical effort is invested and where it is most needed. Further research should clarify the role and value of different report types at each stage of the judicial process- including police investigations, prosecution decisions, and court trials. Such insight could support a more strategic and effective use of forensic medical expertise.

Treating physicians may also provide evidence in court cases. In this study, GPs across all regions were observed to complete standardized forms describing injuries and treatment (referred to as ‘police notes’ in Sect. 3.4). In at least one region, they also appeared to produce injury reports, although the content and scope of these reports were unclear. Previous research has highlighted the evidentiary value of information from treating physicians, while also raising concerns about brevity, handwriting, and the use of medical jargon [28, 29]. 

These issues reflect the current state of forensic medicine education in the Netherlands, which remains limited and lacks standardization. Earlier studies have called for improved training for treating physicians involved in injury assessment—particularly GPs and emergency room doctors [36, 37]. A study by the European Council of Legal Medicine (ECLM) similarly identified significant disparities in undergraduate forensic medicine education across Europe. It recommended harmonizing curricula for consistent, high-quality training [38]. Strengthening training programs and promoting European collaboration could improve the quality and consistency of treating physician’ documentation for legal purposes.

Police and victims may also contribute evidence—through reports, statements, and photographic documentation. In some cases, this may reduce the need for forensic medical involvement. Judges in previous research considered such evidence valuable, but concerns were raised about its quality and objectivity [28, 29]. The extent to which this type of evidence can reliably replace or complement forensic medical expertise requires further investigation. Current research is examining police procedures for injury assessment to help inform future practices [39]. 

### Strengths and limitations

This study has several strengths. It achieved a high response rate, with broad participation from forensic medical departments across the country, ensuring good regional representation. The diverse respondent base and detailed data collection provided multiple perspectives on forensic medical practices, registration, protocols, and operational challenges. Combining quantitative and qualitative analyses strengthened the understanding of current practices and barriers.

Several limitations should also be noted. Non-uniform registration of expert reports limited reliable regional comparisons. A new registration category for expert reports in Formatus (introduced in May 2024) is expected to improve future research. The non-participation of three departments may have introduced minor bias. In addition, the inability to obtain protocols from departments that reported having them limited analysis of collaboration agreements. Finally, the use of self-reported data may have led to recall bias or incomplete responses.

Despite these limitations, the study’s high response rate, broad regional coverage and detailed data provide valuable insights into current forensic medical practices and highlight key areas for improvement.

### Conclusion

In conclusion, significant regional discrepancies exist in the investigation and reporting of non-fatal injury in adults in the Netherlands. These include differences in investigation methods, reporting standards, and the number of reports. These variations are linked to a lack of standardized protocols, inconsistent involvement of forensic doctors, and capacity constraints. Furthermore, the role of treating physicians, police, and victims in providing evidence is not clearly defined.

These findings provide valuable insights into the existing gaps and offer a foundation for enhancing the standardization and effectiveness of forensic medical reporting in non-fatal injury cases. To ensure equitable access to forensic medical expertise, possible measures include national standardization, clear case prioritization for forensic medical involvement, enhanced collaboration, and forensic training for treating physicians.

## Footnotes

1 Abuse (mishandeling) refers to the intentional infliction of bodily harm or physical pain on another person without legal justification, as defined in Articles 300–306 of the Dutch Penal Code. This includes ‘simple’ abuse, abuse resulting in serious bodily injury (SBI) and severe abuse.

2 National Research and Expertise Bureau for Forensic Medical Investigation (Landelijk Onderzoeks- en Expertisecentrum Forensisch Medisch Onderzoek, LOEF).
