# Correction to: Forensic medical reporting of non-fatal injuries in criminal cases in the Netherlands: a mixed-methods analysis of regional practices

**DOI:** 10.1007/s00414-026-03719-y

**Published:** 2026-01-23

**Authors:** Maartje L. Goudswaard, Joyce N. Cuijpers, Manon Ceelen, Kim H. de Bruin, Udo J. L. Reijnders, H. Ibrahim Korkmaz, Dionne S. Kringos

**Affiliations:** 1 Department of Forensic Medicine and Medical Advice, Public Health Service Amsterdam, Amsterdam, the Netherlands; 2 Department of Public and Occupational Health, Amsterdam UMC, University of Amsterdam, Amsterdam, the Netherlands; 3 Department of Plastic Reconstructive & Hand Surgery, Amsterdam Movement Sciences (AMS) Institute, Molecular Cell Biology & Immunology, Amsterdam Infection and Immunity (AII) Institute, Amsterdam UMC, Location VUmc, Amsterdam, the Netherlands; 4 Alliance of Dutch Burn Care, Beverwijk, the Netherlands; 5 Burn Center and Department of Plastic and Reconstructive Surgery, Red Cross Hospital, Beverwijk, the Netherlands; 6 Quality of Care, Amsterdam Public Health Research Institute, Amsterdam, the Netherlands

**Correction to: International Journal of Legal Medicine**

10.1007/s00414-025-03678-w

In the original version of the manuscript, both full first names of the authors and their corresponding initials are present, causing duplication, and the initial of one author was missing.

The correct names are:

**Maartje L. Goudswaard**

**Joyce N. Cuijpers**

**Manon Ceelen**

**Kim H. de Bruin**

**Udo J.L. Reijnders**

**H. Ibrahim Korkmaz**

**Dionne S. Kringos**

Additionally, Figures 2-4 in the Springer Link are duplicated. The first figures displayed are the correct figures and should be included. The duplicated figures should be deleted.

The original article has been corrected.

